# Drug reaction with eosinophilia and systemic symptoms syndrome (DRESS) syndrome associated with azithromycin presenting like septic shock: a case report

**DOI:** 10.1186/1752-1947-8-332

**Published:** 2014-10-08

**Authors:** Narin Sriratanaviriyakul, Lam-Phuong Nguyen, Mark C Henderson, Timothy E Albertson

**Affiliations:** 1 Division of Pulmonary, Critical Care, and Sleep Medicine, University of California, Davis, 4150 V Street, Sacramento, CA 95817, USA; 2 Department of Internal Medicine, University of California, Davis, 4150 V Street, Sacramento, CA 95817, USA; 3 VA Northern California Health Care System, 10535 Hospital Way, Mather, CA 95655, USA

**Keywords:** DRESS syndrome, Drug allergy, Drug-induced hypersensitivity syndrome, Eosinophilia, Erythroderma, Azithromycin hypersensitivity

## Abstract

**Introduction:**

Drug reaction with eosinophilia and systemic symptoms syndrome is a potentially life-threatening cutaneous hypersensitivity reaction characterized by extensive mucocutaneous eruption, fever, hematologic abnormalities including eosinophilia and/or atypical lymphocytosis, and extensive organ involvement. The drugs most often responsible for causing drug reaction with eosinophilia and systemic symptoms syndrome are anticonvulsants, antimicrobial agents and antipyretic or anti-inflammatory analgesics. Although azithromycin is widely prescribed in clinical practice, serious cutaneous reactions from this agent have been rarely described. We report the first adult case of drug reaction with eosinophilia and systemic symptoms syndrome associated with azithromycin.

**Case presentation:**

A 44-year-old previously healthy Caucasian man with history of tobacco use presented to his primary care physician with fever and productive cough. He was prescribed azithromycin, promethazine hydrochloride and dextromethorphan hydrobromide syrup. One week later, he developed a blistering erythematous rash over both hands, which over the next two weeks spread to involve nearly his entire body surface, sparing only his face. He was admitted to an outside hospital with signs of systemic inflammatory response syndrome and severe sepsis, presumably from a skin infection. Despite aggressive therapy he deteriorated, with worsening diffuse erythema, and was transferred to our institution. He developed multiple organ failure requiring ventilatory and hemodynamic support. Pertinent laboratory studies included a leukocytosis with a white blood cell count of 17.6×10^9^/L and 47% eosinophils. A skin biopsy showed evidence of spongiotic lichenoid dermatitis with eosinophils and neutrophils, compatible with a systemic drug-induced hypersensitivity reaction. Our patient was started on high-dose steroids and showed dramatic improvement within 48 hours.

**Conclusions:**

We report the first adult case of drug reaction with eosinophilia and systemic symptoms syndrome associated with azithromycin exposure. Clinicians should be aware of this potentially devastating complication from this commonly prescribed medication.

## Introduction

Cutaneous hypersensitivity reactions range in severity, from mild reactions to severe cutaneous adverse reactions, or SCARs. SCARs include a spectrum of disease from erythema multiforme (EM) to drug reaction with eosinophilia and systemic symptoms (DRESS) to toxic epidermal necrolysis (TEN), the most serious form of drug reaction. Mild adverse cutaneous reactions to drugs are common, occurring in up to 2% to 3% of all hospitalized patients [1]. Of all the cutaneous adverse reaction, 2% of cases are severe and very few are fatal [2].

DRESS syndrome is a potentially life-threatening reaction characterized by extensive mucocutaneous eruption, fever, hematologic abnormalities including eosinophilia and/or atypical lymphocytosis, and extensive internal organ involvement [3]. Long-term sequelae and multiple organ involvement include myocarditis or pericarditis, interstitial nephritis, necrotizing granulomatous vasculitis, encephalitis or meningitis, as well as shock and respiratory distress syndrome. The drugs most often responsible are anticonvulsants (mostly aromatase derivatives), antimicrobial agents, particularly penicillin and sulfonamide-based agents, and antipyretic/anti-inflammatory analgesics [2,4]. DRESS syndrome has a latency onset, characteristically occurring within two to six weeks after initial exposure to the inciting drug. In a few reported cases, the syndrome becomes persistent despite discontinuation of the culprit drug [5].

Azithromycin is widely prescribed for acute respiratory tract infection. The majority of adverse events from azithromycin are mild to moderate in severity and related to the gastrointestinal tract. Drug rash is observed in approximately 6% of cases [6]. However, severe cutaneous reactions have been rarely reported and none have developed DRESS syndrome according to our literature search. Here we report the potential first case of DRESS syndrome associated with azithromycin.

## Case presentation

**Figure 1:**
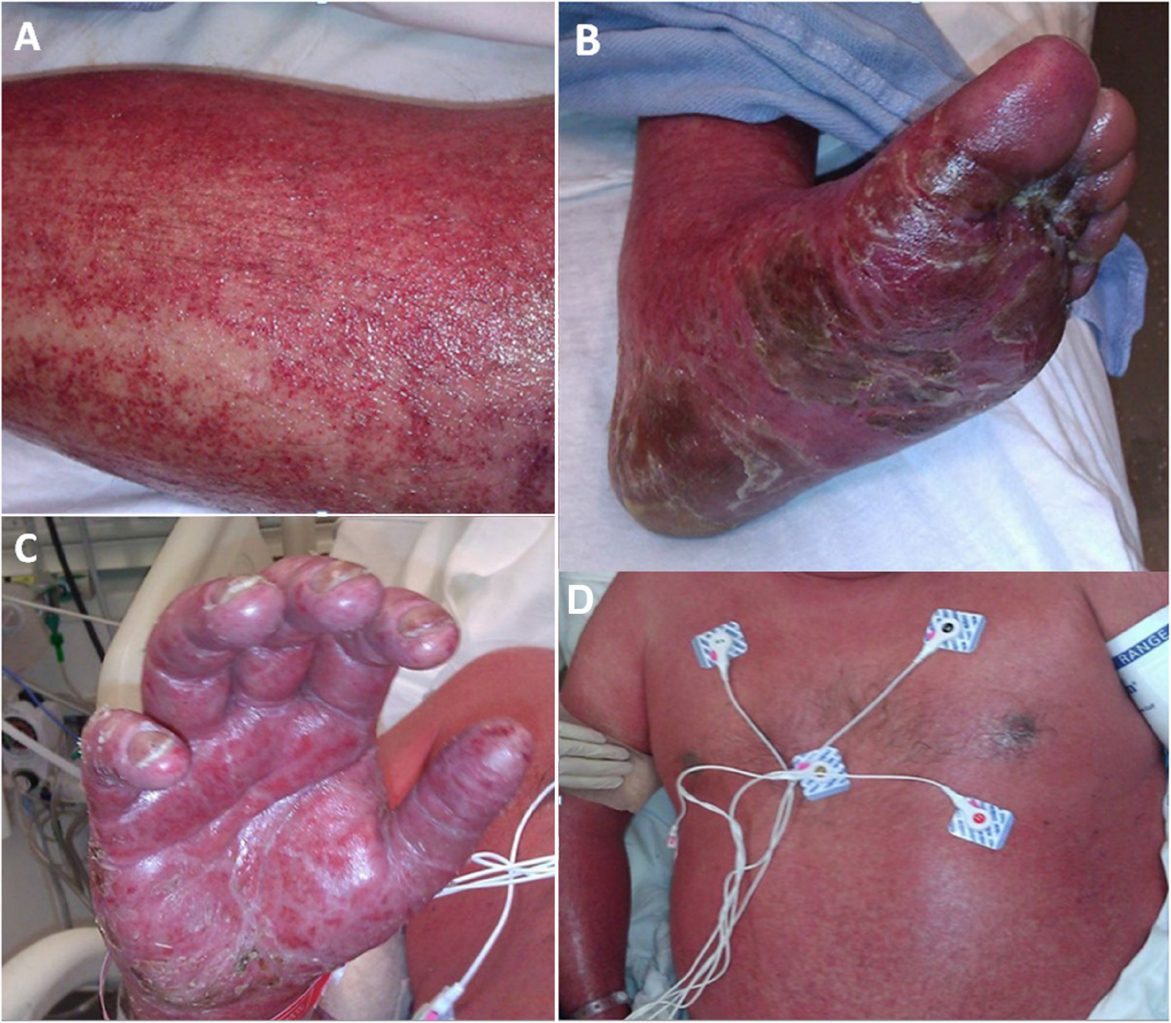
**(A) Left leg, (B) left foot, (C) right hand and (D) trunk with ill-defined coalescing erythema from head to toe with diffuse pinpoint petechiae.** Keratotic desquamation of palms and soles.

A 44-year-old otherwise healthy Caucasian man, with a past medical history of anxiety and 15 pack year tobacco use, initially presented to his primary care physician with several days onset of fevers, congestion and cough. He was prescribed azithromycin, promethazine hydrochloride and dextromethorphan hydrobromide syrup. One week later, the patient developed an acute diffuse blistering morbilliform erythematous rash involving bilateral hands, which was extremely pruritic and associated with high-grade fever. Within two weeks, the rash spread to involve nearly his entire body surface, sparing only his face. He presented to an outside hospital with persistent fever, rash, and hypotension consistent with systemic inflammatory response syndrome. He was treated for severe sepsis and presumptive superimposed skin infection with vancomycin and aztreonem, given his history of penicillin allergy. Despite aggressive therapy, the patient rapidly deteriorated with worsening skin involvement, and was subsequently transferred to our institution for higher level care.Upon arrival, his vital signs were temperature of 38.2°C, blood pressure of 95/20mmHg, heart rate 68 beats/min, respiratory rate 20 breaths/min, and oxygen saturation 95% on 2L nasal canula. Shortly thereafter, our patient became hypotensive despite aggressive fluid resuscitation and was started on norepinephrine. Our patient was subsequently intubated due to worsening level of consciousness. A physical examination revealed an ill-appearing sedated man with evidence of generalized ill-defined coalescing erythema and diffuse pinpoint petechiae. A pulmonary examination was notable for bilateral crackles and diffuse rhonchi and a cardiovascular examination was normal except for tachycardia. There was no lymphadenopathy. A detailed skin examination (Figure 1) revealed keratotic desquamation of his palms and soles. His lips and oral mucosa were dry with some cracking of vermilion lips but no bullae. His tongue was diffusely red. His conjunctivae were injected. There were no vesicles, bullae, or target lesions seen. The Nikolsky sign was negative.

**Figure 2:**
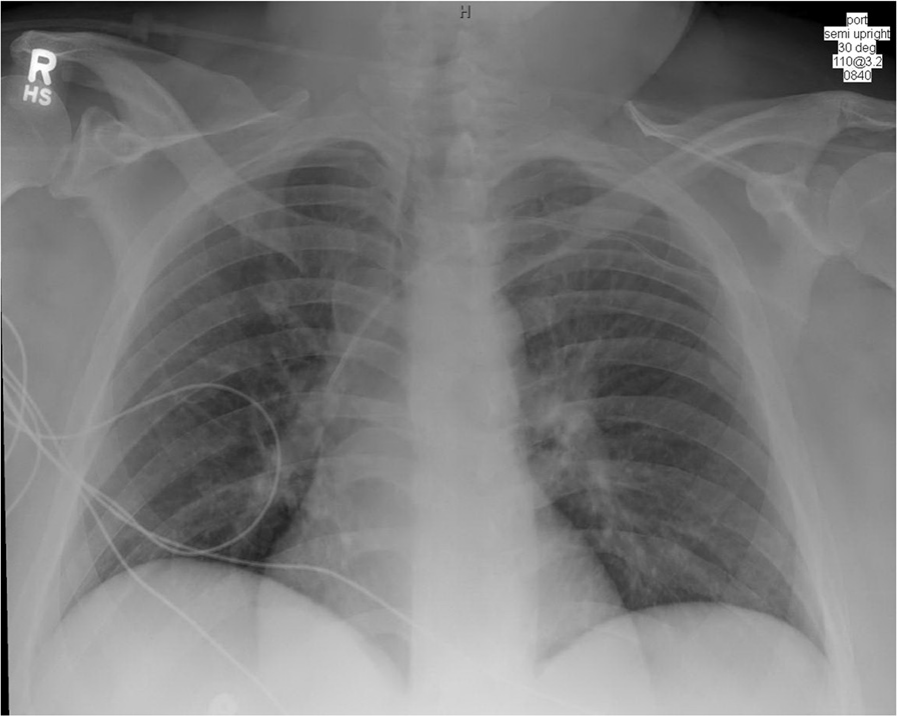
Chest X-ray showing pulmonary edema.

Laboratory studies revealed leukocytosis with a white blood cell count of 17.6×10^9^/L with 47% eosinophils, 33% neutrophils, 5% bands, and 12% lymphocytes as well as thrombocytopenia of 117×10^9^/L. No atypical lymphocytes were seen on the peripheral blood smear or manual differential. His blood chemistry test results were notable for blood urea nitrogen of 21.4mmol/L, serum creatinine 229μmol/L, albumin 21g/L, alkaline phosphatase 188μg/L, aspartate aminotransaminase 58IU/mL, and alanine aminotransferase 37IU/mL. Serological test results including HIV titer, leptospira antibody, and streptolysin O antibody were all nonreactive. His blood cultures, urine culture, herpes simplex wound culture, as well as varicella zoster wound cultures were all unremarkable. Coccidioides serology was negative. His chest X-ray was consistent with pulmonary edema (Figure 2). Results from a diagnostic bronchoscopy showed evidence of diffuse mucosal hyperemia from the trachea through the subsegmental bronchi but no sloughing. Bronchial washing studies including bacterial, viral and fungal cultures, as well as pneumocystis jiroveci DFA, were all unremarkable.

**Figure 3:**
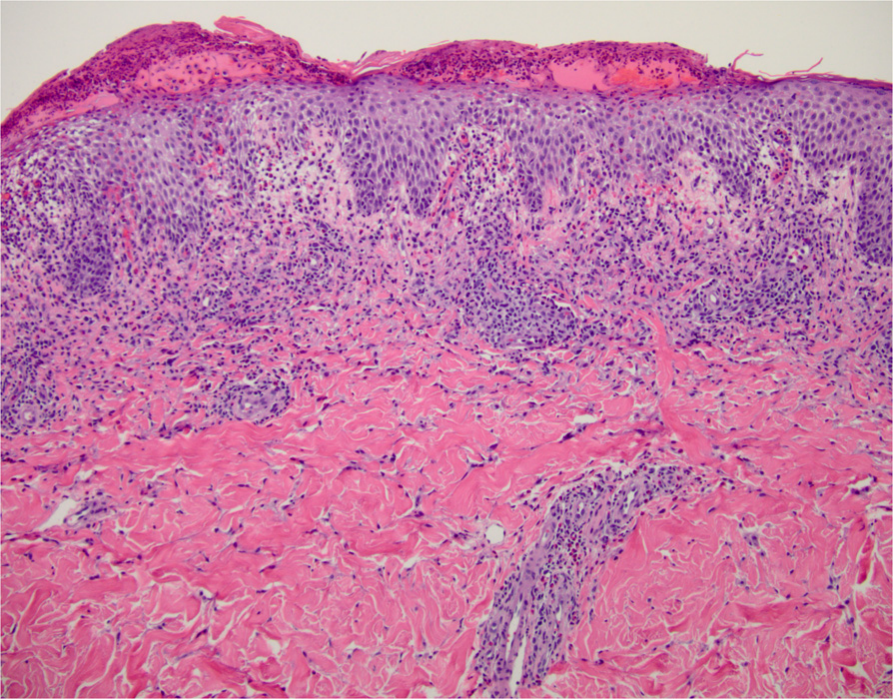
**Spongiotic and interface dermatitis with eosinophils (hematoxylin and eosin, ×200).**

**Figure 4:**
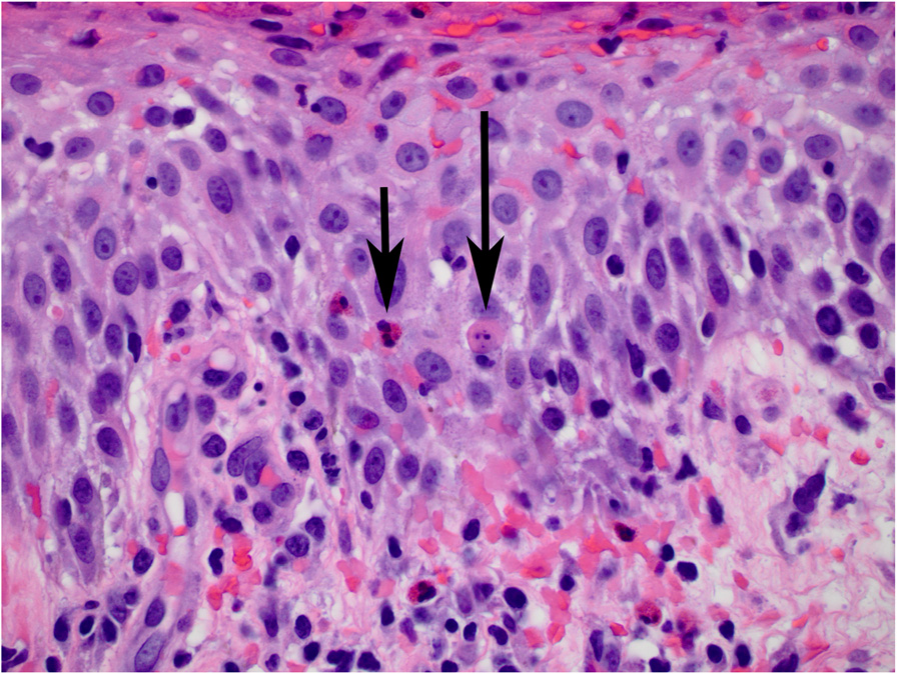
**Eosinophilic spongiosis (short arrow) and a necrotic/apoptotic keratinocyte (long arrow).** (Hematoxylin and eosin, ×600).

**Figure 5:**
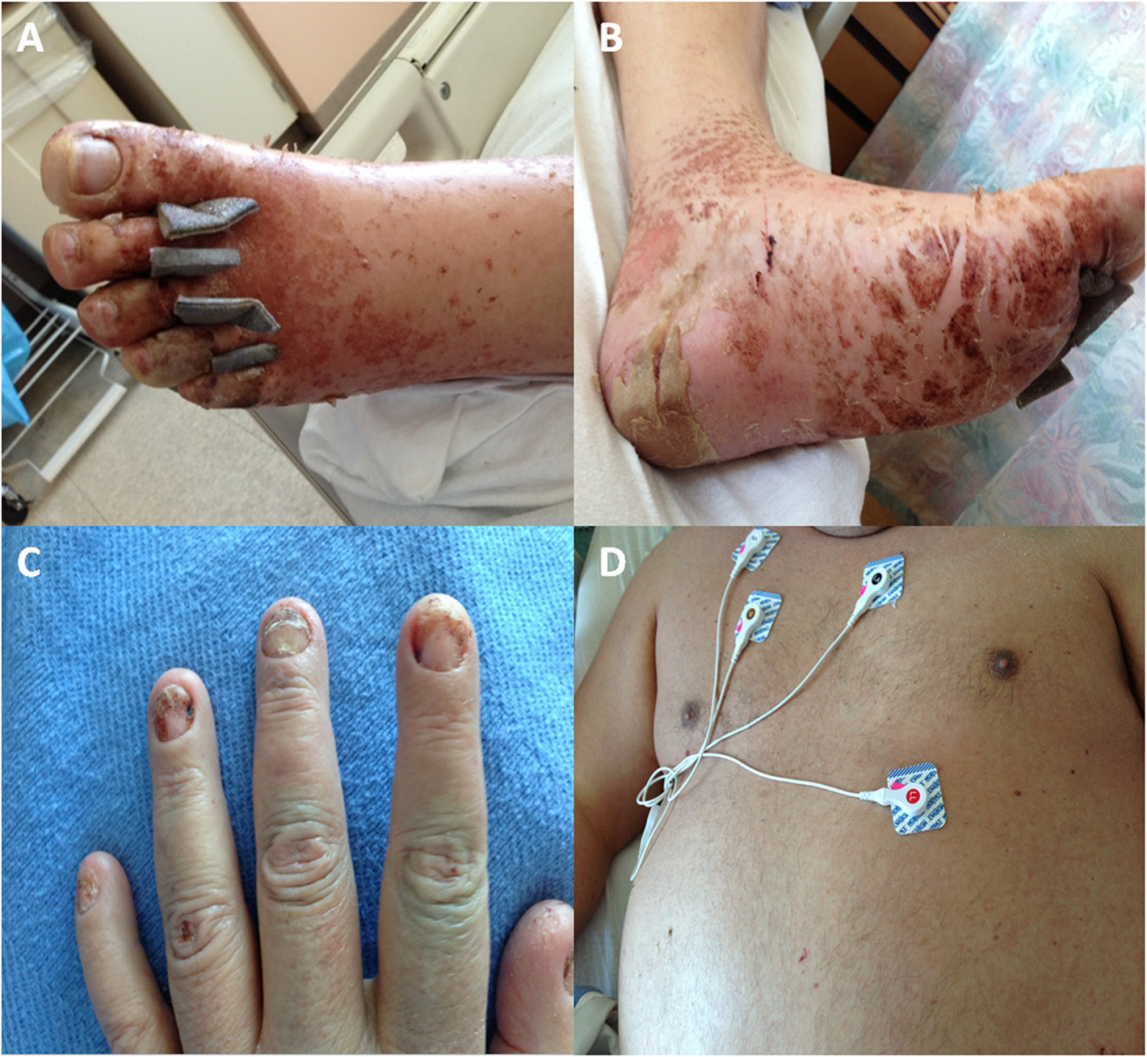
**(A and B) Our patient’s left foot with residual superficial erythematous rash. (C)** Inflammation extending into bilateral hands and involving fingernails. **(D)** Faint erythematous rash involving the chest, abdomen and trunk.

Despite an exhaustive workup noted above and consultation with dermatology, infectious disease, ophthalmology, pulmonary and burn specialists, no clear etiology was identified. Over the next several days, he continued to worsen despite broad-spectrum antibiotics including vancomycin, meropenem and clindamycin. He developed low-grade disseminated intravascular coagulation (DIC), worsening leukocytosis, worsening renal failure as well as persistent hypotension requiring high levels of norepinephrine.On hospital day 4, results from his skin biopsy showed evidence of spongiotic lichenoid dermatitis with eosinophils and neutrophils (Figures 3 and 4), compatible with systemic drug hypersensitivity reaction. No evidence of vasculitis was seen on histopathology. Our patient was started on high-dose steroids and all antibiotics were discontinued, given no convincing evidence of infection. He was weaned off norepinephrine and extubated shortly thereafter. His fever resolved and his generalized body rash dramatically improved over the course of 48 hours with supportive treatment (Figure 5). Our patient subsequently made a full recovery after an additional week of rehabilitation and was discharged home.

## Discussion

DRESS syndrome is a severe drug reaction with a mortality rate up to 10% despite accurate diagnosis and removal of the offending agent. Diagnosis is challenging and often delayed, given its variable presentation. Currently, there are no accurate diagnostic criteria for DRESS but it is a syndrome diagnosed by exclusion. The main clinical features are rash, fever, hematological abnormalities and organ involvement, all of which can be nonspecific. The RegiSCAR scoring system has been recently developed to delineate each of severe cutaneous adverse reactions (SCARs) as distinct entities [7]. The RegiSCAR scoring system was designed to grade suspected cases of DRESS as ‘no’ , ‘possible’ , ‘probable’ or ‘definite’. DRESS is considered as definite in our case given that other causes of febrile eruption with eosinophilia and liver involvement were ruled out. We suspect that azithromycin is likely the culprit medication in this scenario. Promethazine may also produce drug reactions, but the association with DRESS syndrome has not been reported. There are two available methods of confirming the culprit drug of DRESS syndrome: skin patch tests and lymphocyte transformation tests [8]. Unfortunately, neither is widely accepted or used.

According to one recent review of 172 DRESS cases, there are 44 drugs associated with DRESS [9]. Of these, the most frequently reported were carbamazepine, allopurinol, sulfasalazine, phenobarbital, lamotrigine and nevirapine. Azithromycin has not been shown to cause DRESS in adults. Thus far, there have been only two reported pediatric cases of DRESS associated with azithromycin [10,11]. One of the cases was also associated with Epstein-Barr virus (EBV) primary infection. Pursnani *et al.* reported a case of fulminant myocarditis in an adult associated with azithromycin [12], which was presumed to be DRESS syndrome; however, the RegiSCAR scoring system was not reported.

The pathophysiology of DRESS syndrome has not been fully elucidated. Different mechanisms have been implicated, including detoxification defects leading to reactive metabolite formation and subsequent immunological reactions, slow acetylation, and reactivation of human herpes, including EBV and human herpesvirus (HHV)-6 and -7 [9]. The association of HHV-6 infection or reactivation and severe DRESS syndrome has also been reported [13]. It is postulated that HHV-6 may interfere with some of the enzymes responsible for drug detoxification. The virus itself could also be responsible for the skin lesions and some of the visceral involvement [13]. The recent studies suggest that HHV-6 reactivation activates CD8+ T lymphocytes causing it to secrete cytokines [14].

There are no consensus guidelines on the management of DRESS syndrome. The mainstay of treatment is discontinuing the culprit drug. The use of systemic steroids is controversial including the route of administration and dosing [9].

## Conclusions

In summary, we report the first adult case of definite DRESS syndrome associated with azithromycin exposure. Early recognition and prompt removal of the culprit agent is the treatment of choice.

## Consent

Written informed consent was obtained from the patient for publication of this case report and any accompanying images. A copy of the written consent is available for review by the Editor-in-Chief of this journal.

## Abbreviations

DIC: disseminated intravascular coagulation; DRESS: drug reaction with eosinophilia and systemic symptoms; EBV: Epstein-Barr virus; EM: erythema multiforme; HHV: human herpesvirus; SCAR: severe cutaneous adverse reactions; TEN: toxic epidermal necrolysis.

## Competing interests

The authors declare that they have no competing interests.

## Authors’ contributions

NS, LN, MH and TA collected patient data and administered therapy. NS wrote the manuscript. LN, MH and TA revised and edited the manuscript. All authors read and approved the final manuscript.

## References

[B1] Bigby M Jick S Jick H Arndt K Drug-induced cutaneous reactions. A report from the Boston Collaborative Drug Surveillance Program on 15,438 consecutive inpatients, 1975 to 1982 JAMA 1986 256 3358 3363 10.1001/jama.1986.03380240052027 2946876

[B2] Alanko K Stubb S Kauppinen K Cutaneous drug reactions: clinical types and causative agents. A five-year survey of in-patients (1981-1985) Acta Derm Venereol 1989 69 223 226 2566225

[B3] Walsh SA Creamer D Drug reaction with eosinophilia and systemic symptoms (DRESS): a clinical update and review of current thinking Clin Exp Dermatol 2011 36 6 11 10.1111/j.1365-2230.2010.03967.x 21143513

[B4] Wang F Li Y Mo Y Shen C Yang L Zhang X Cutaneous adverse drug reactions: an 8-year retrospective study on hospitalized patients in Southern China Indian J Dermatol Venereol Leprol 2012 78 488 490 10.4103/0378-6323.98082 22772622

[B5] Bocquet H Bagot M Roujeau JC Drug-induced pseudolymphoma and drug hypersensitivity syndrome (Drug Rash with Eosinophilia and Systemic Symptoms: DRESS) Semin Cutan Med Surg 1996 15 250 257 10.1016/S1085-5629(96)80038-1 9069593

[B6] Harris JA Kolokathis A Campbell M Cassell GH Hammerschlag MR Safety and efficacy of azithromycin in the treatment of community-acquired pneumonia in children Pediatr Infect Dis J 1998 17 865 871 10.1097/00006454-199810000-00004 9802626

[B7] Kardaun SH Sidoroff A Valeyrie-Allanore L Halevy S Davidovici BB Mockenhaupt M Roujeau JC Variability in the clinical pattern of cutaneous side-effects of drugs with systemic symptoms: does a DRESS syndrome really exist? Br J Dermatol 2007 156 609 611 10.1111/j.1365-2133.2006.07704.x 17300272

[B8] Husain Z Reddy BY Schwartz RA DRESS syndrome: part II. Management and therapeutics J Am Acad Dermatol 2013 68 709 e1-9; quiz 718-720 23602183 10.1016/j.jaad.2013.01.032

[B9] Cacoub P Musette P Descamps V Meyer O Speirs C Finzi L Roujeau JC The DRESS syndrome: a literature review Am J Med 2011 124 588 597 10.1016/j.amjmed.2011.01.017 21592453

[B10] Bauer KA Brimhall AK Chang TT Drug reaction with eosinophilia and systemic symptoms (DRESS) associated with azithromycin in acute Epstein-Barr virus infection Pediatr Dermatol 2011 28 741 743 10.1111/j.1525-1470.2011.01558.x 22010986

[B11] Schmutz JL Trechot P [DRESS associated with azithromycin in a child] Ann Dermatol Venereol 2013 140 75 10.1016/j.annder.2012.09.006 23328369

[B12] Pursnani A Yee H Slater W Sarswat N Hypersensitivity myocarditis associated with azithromycin exposure Ann Intern Med 2009 150 225 226 10.7326/0003-4819-150-3-200902030-00027 19189924

[B13] Ichiche M Kiesch N DeBels D DRESS syndrome associated with HHV-6 reactivation Eur J Intern Med 2003 14 498 500 10.1016/j.ejim.2003.09.004 14962704

[B14] Picard D Janela B Descamps V D’Incan M Courville P Jacquot S Rogez S Mardivirin L Moins-Teisserenc H Toubert A Benichou J Joly P Musette P Drug reaction with eosinophilia and systemic symptoms (DRESS): a multiorgan antiviral T cell response Sci Transl Med 2010 2 46ra62 20739682 10.1126/scitranslmed.3001116

